# Classification of breast carcinomas according to gene expression profiles

**Published:** 2013-03-25

**Authors:** L Moldovan, A Mitroi, CM Petrescu, M Aschie

**Affiliations:** * Clinical Studies Department, Panduri Hospital of Bucharest; ** Pathology Department, Clinical Emergency County Hospital of Constanta; *** "Carol Davila" University of Medicine and Pharmacy Bucharest, National School of Political and Administrative Studies Bucharest

**Keywords:** breast carcinoma, gene expression profile, microarray

## Abstract

Breast carcinomas represent an important health problem. Understanding the development of breast cancer from precursor is critical for clinical treatment and prevention, however little is known about the molecular events involved in the progression to cancer. The advent of gene expression microarray technology provides a new powerful tool to assist in the determination of diagnosis, prognosis and treatment. In this paper, we present the recent DNA microarray studies that describe how gene expression profiling is being used to classify specimens of breast carcinomas based on molecular properties of the tumor and to identify gene expression patterns related to clinical outcome. In present, data are available that show that gene expression profiles can be used to distinguish cell type-specific gene clusters (stromal, epithelial, mesenchymal and proliferation status) and to classify breast tumors as basal-like, luminal-like, ERBB2 overexpressing and normal breast-like. Profiles associated with good prognosis and poor prognosis of young axillary node negative patients have been identified. The microarray technology will become in the near future a molecular complement to histopathology and immnuhistochemistry.

## Introduction

Breast carcinomas represent an important health problem that has proven to be a challenge for clinical and basic science research because of cellular heterogeneity. Beside, the great number of genes involved in controlling cell physiology complicates the accurate prognosis of clinical behavior of breast cancer. 

Expression profiles refer to the process of measuring the expression of thousands of genes simultaneously in a given tissue sample. The resulting patterns of gene expressions reflect the molecular basis of the tumor phenotype and can be used for tumor comparisons and classification. The advent of gene expression microarray technology provides a powerful tool to assist in the determination of diagnosis, prognosis and treatment.

Understanding the development of breast cancer from precursor is critical for clinical treatment and prevention, however little is known of the molecular events involved in the progression to cancer. Currently, available prognostic and predictive markers are not sufficient for the accurate determination of risk for many breast cancer patients. Thus, it is necessary the discovery of new molecular markers which obviously be of value in accurate prediction of clinical outcome and in individualizing therapy.

 A numbers of recent studies have reported the use of gene expression arrays to identify groups of co-expressed genes, to characterize genes by their expression profiles over a set of breast carcinoma samples, and to characterize molecular signatures of breast carcinomas [**1**]. In this paper, we present the recent studies that describe how gene expression profiling is being used to classify specimens of breast carcinomas based on properties of the tumor, such as expression of ER and ERBB2, as well as p53 mutation status, to identify gene expression patterns related to clinical outcome and to predict therapeutic groups responsive to hormonal and chemotherapeutic agents. 

## Materials and methods

 To characterize gene expression patterns in human breast cancer, investigators have studied array profiles of breast epithelial cell cultures, breast cancer cell lines and primary human breast carcinoma. 

The most used method of tumor expression profile is cDNA microarray hybridization-based. In this technique, mRNA is extracted from the tumor sample, converted by reverse transcription to cDNA, and then hybridized to a DNA microarray. Each feature on the array is referred to as a “probe" and the mixture derived from the sample is the “target" [**2**]. DNA microarrays are either nylon membranes, glass slides, or synthetic “chips", to which are attached nucleic acid probes as cDNA clones or cDNA clone-specific oligonucleotides corresponding to hundreds to tens thousands of genes. 

 A fluorochrome can be incorporated directly, coupled to a reactive group or used in secondary detection. For two-color hybridization, it is necessary to select a reference sample. In principle, the primary requirement o this material is a similar pattern of gene expression to the tumors for which it will be compared. If many genes, which are strongly expressed in the tumors, are expressed in the reference sample at near background levels, then the sample-to-reference ratio will be unreliable [**2**]. This requirement for similar expression may be difficult to meet. One approach is to use a related cancer cell line or a pool of cell lines [**2**]. It is important to carry out a test hybridization to determine the suitability of a reference RNA before proceeding. Normal tissue truly representing the cancer progenitor cell is not generally available in sufficient quantity to use as a reference. The exception may be in those situations in which micro dissected material will be amplified.

 After the hybridization, a fluorescence image of the microarray is obtained with the scanning device, and the image file is processed with the feature extraction software, which converts the raw image to numerical data corresponding to the level of fluorescence in each channel. There are commercial instruments and software packages for the purpose that performs well. 

The statistical analysis of gene expression data from microarray studies of breast carcinoma follow the processes outlined in **Table 1**.

**Table 1:** Statistical analysis of gene expression data

Preprocessing of each array
- Image analysis
- Quality assessment
- Normalization
- Diagnostic plots
Selection of array sets and genes to be include in analysis
Unsupervised analysis methods
- Identification of clusters of samples with similar expression signatures
- Identification of clusters of genes with similar expression profiles
Supervised analysis methods
- Univariate single gene comparisons among groups of samples
- Multivariate multiple gene comparisons among groups of samples
- Prediction and validation of group membership for individual samples.

## Results of array studies

*I. Examination of Expression Profiles of Breast Cultured Cells and Primary Tumors*

 Ross et al. used cDNA microarrays to classify cell lines according to their tissue origin [**3**]. They performed molecular classification of 60 cancer cell lines derived from tumors of a variety of tissues and organs using arrays of 9703 human cDNAs. They showed a consistent relationship between gene expression patterns and the tumors tissue of origin. Based on the gene expression profiles, Ross et al. identified groups of genes they considered to represent epithelial, mesenchymal, stromal, and proliferation clusters [1,3]. By comparing the gene expression signatures of two breast cancer specimens to a normal tissue specimen and to cultured breast cancer cell lines, they were able o distinguish between different cellular counterparts of breast tumors. Expression of keratin 8 and keratin 19 in the estrogen positive (ES+) breast cancer cell lines suggested that these cells had originated from luminal epithelial cells [**1**]. On the other hand, stromal-like cell lines had high levels of expression of collagen genes (COL3A1, COL5A1, COL6A1) and a smooth muscle cell marker (TAGLN), which are characteristic of stromal counterparts.

 Su et al. also classified human carcinomas by analyzing 100 primary carcinomas from 10 diverse tissues of origin including breast. Using expression arrays of 12.533 oligonucleotides, they identified highly restricted tumor-specific expression patterns and demonstrated the feasibility of predicting the tissue of origin of a carcinoma based on expression patterns [**4**].

 Bertucci et al. studied genes expression of 34 primary breast carcinomas using 176 gene arrays [**5**]. Hierarchical clustering was performed on the tumors and genes, and they identified two subgroups of tumors with distinct clinical outcomes. They also compared the gene expression between normal tissue and tumor specimens, between ER- and ER+ tumors, and between ANN tumors and tumors with involved lymph node. The transcription factor GATA3 showed high levels of expression in ER+ tumor group. MYB proto-oncogene, X-box binding protein 1, p53 and insulin-like growth factor 2 were also differentially expressed in ER+ compared with ER- tumors [**1**]. They also found a correlation between ERBB2 expression and nodal status.

*II. Tumor Classification Based o Expression Profiles*

*II.1 Tumor Classification Related to ERBB-2 Expression*

 The normal human mammary gland contains two types of epithelial cells that can be distinguished by immunohisochemical staining:

 - the basal-myoepithelial cells, which express keratin 5/6 and 17;

 - the luminal cells which express keratin 8/18 [**6**].

 To develop a system for classifying tumors o the basis of their expression pattern, Perou et al. [**7**] chose a subset of 496 genes (intrinsic gene subset) that showed a greater variation in expression pattern between different tumors, to use as the basis for cluster analysis. Thus, they clustered the breast tumors in four main groups, which they describe as:

 - ER+/luminal-like,

 - Basal-like,

 - ERBB2+,

 - Normal breast-like.

 The ER+ tumor group had high levels of expression o genes characterized as the luminal profile, including GATA3, an stained with antibodies against luminal cell keratins 5/6 and 17, or were in the group that had high expression of ERBB2 and related genes [**1,7**].

 Sorlie et al. analyzed a total of 78 breast carcinomas, 3 fibroadenomas and 4 normal breast tissue samples using the intrinsic gene subset as basis for tissue classification [**8**]. Clustering analysis separated the tumors into two main branches. The first one contained previously defined gene subgroups (basal-like, ERBB2+, and normal breast-like) and in other branch the luminal/ER+ group was divided into three groups (luminal subtypes A, B, and C) [**1,8**].

*II.2 Expression Profiles Related to p53 Mutation Status*

 Mutation in the p53 gene is common in breast cancer and has been found to be of prognostic significance in some studies [**1**]. Sorlie et al. examined the correlation of p53 status and tumor subclass in 69 tumors of their set, 30 of which had mutations in the p53 gene. They found a difference in the distribution of p53 mutations among subclasses. The ERBB2+ and basal subclasses had p53 mutation in 71 and 82% of tumors, whereas the luminal subtype A contained p53 mutation only in 13% of the cases. Luminal subtype C presented the same features with the ERBB2 positive and basal-like subclasses, including p53 mutations in approx 80% of tumors [**8**].

*III. Expression Profile Related to Clinical Outcome and Disease-Free Survival.*

 A study effectuated by Sorlie et al. from 85 tissue specimens with breast carcinomas, which included 49 cases with locally advanced disease but not distant metastases, showed significantly different outcomes among the patients belonging to subgroups of tumors identified in cluster analysis [**8**]. The basal-like and ERBB2+ types were associated with shortest survival times. There was a significant difference in the outcome for patients in the luminal group, with the luminal C tumors having the worst outcome. Because the luminal C subgroup exhibits molecular similarities to those of the ERBB2+ and basal-like subtypes, it seems that overexpressing of a common set of genes may be associated with poor outcome.

 In a recent study, van’t Veer et al. performed the microarray expression analysis on tumors from 98 young breast cancer patients (age at diagnosis <55 year) [**9**]. They analyzed 34 tumors from axillary node negative patients who developed metastases within 5 years, 44 tumors from axillary node negative patients who were disease-free after a period of at least 5 year, 18 BRCA1 mutation carriers and 2 BRCA2 mutation carriers.

 Two distinct clusters were interpreted as representing good prognosis and poor prognosis tumors according to the disease free survival status of the sporadic tumors in the cluster. The authors also investigated the association of these data with the ER status of the patients. The majority of the ER- tumors clustered together in the poor prognosis branch of the tumor cluster [**9**]. A gene cluster containing the ER gene and genes that are co-regulated with ER were found to have low expression in the poor prognosis tumor group, while a second gene cluster containing genes that represent lymphocytic infiltration was found to have higher expression. Sixteen of eighteen BRCA1 carriers were also in the poor prognosis group together with ER- tumors and tumor with lymphocytic infiltration [**9**]. 

 The prognosis classifier correctly predicted the outcome in the 83% o the cases. Genes including cyclin E2, MMP9, MMP1 and others genes involved in cell cycle, invasion, metastasis, angiogenesis and signal transduction were significantly up-regulated in tumors with poor prognosis [**1**]. Other genes, like ERBB2, ER and cyclin D1, that may have been expected to be associated with prognosis were not. It should be noted that expression of ERBB2 as a prognostic marker is derived primarily from studies using immunohistochemistry and DNA copy number, not mRNA levels. 

## Discussion

The results to date of gene expression arrays are valorous. Data are already available that show that expression profiling can be used to distinguish cell type-specific gene clusters (stromal, epithelial, mesenchymal and proliferation status) and to classify breast tumors as basal-like, luminal-like, ERBB2 overexpressing and normal breast-like. Gene expression profiles have been characterized and profiles associated with good prognosis and poor prognosis groups of young axillary node negative patients have been identified.

The microarray technology will provide in the near future a molecular complement to histopathology and immnuhistochemistry. In present are developed sensitive methods which will permit the examination of biological specimens containing a limited number of cells (such as premalignant breast lesions) and aid in the determination of the molecular events involved in the development and progression of breast neoplasia.
